# Plasma autoantibodies to glial fibrillary acidic protein (GFAP) react with brain areas according to Braak staging of Parkinson’s disease

**DOI:** 10.1007/s00702-022-02495-4

**Published:** 2022-04-01

**Authors:** Eva Gschmack, Camelia-Maria Monoranu, Hecham Marouf, Sarah Meyer, Lena Lessel, Raja Idris, Daniela Berg, Walter Maetzler, Frank Steigerwald, Jens Volkmann, Manfred Gerlach, Peter Riederer, Eleni Koutsilieri, Carsten Scheller

**Affiliations:** 1 Institute of Virology and Immunobiology, University of Würzburg, Versbacher Strasse 7, 97078 Würzburg, Germany; 2 Institute of Pathology, Department of Neuropathology, University of Würzburg, Comprehensive Cancer Center (CCC) Mainfranken, Würzburg, Germany; 3 Department of Neurology, Christian-Albrechts-University, Kiel, Germany; 4 Department of Neurodegeneration, Hertie Institute for Clinical Brain Research, University of Tübingen, Tübingen, Germany; 5 Department of Neurology, University Hospital of Würzburg, Würzburg, Germany; 6 Department of Child and Adolescent Psychiatry, Psychosomatics and Psychotherapy, University Hospital of Würzburg, Centre of Mental Health, Würzburg, Germany

**Keywords:** Parkinson, GFAP, Autoantibodies, Braak

## Abstract

Idiopathic Parkinson’s disease (PD) is characterized by a progredient degeneration of the brain, starting at deep subcortical areas such as the dorsal motor nucleus of the glossopharyngeal and vagal nerves (DM) (stage 1), followed by the coeruleus–subcoeruleus complex; (stage 2), the substantia nigra (SN) (stage 3), the anteromedial temporal mesocortex (MC) (stage 4), high-order sensory association areas and prefrontal fields (HC) (stage 5) and finally first-order sensory association areas, premotor areas, as well as primary sensory and motor field (FC) (stage 6). Autoimmunity might play a role in PD pathogenesis. Here we analyzed whether anti-brain autoantibodies differentially recognize different human brain areas and identified autoantigens that correlate with the above-described dissemination of PD pathology in the brain. Brain tissue was obtained from deceased individuals with no history of neurological or psychiatric disease and no neuropathological abnormalities. Tissue homogenates from different brain regions (DM, SN, MC, HC, FC) were subjected to SDS-PAGE and Western blot. Blots were incubated with plasma samples from 30 PD patients and 30 control subjects and stained with anti-IgG antibodies to detect anti-brain autoantibodies. Signals were quantified. Prominent autoantigens were identified by 2D-gel-coupled mass spectrometry sequencing. Anti-brain autoantibodies are frequent and occur both in healthy controls and individuals with PD. Glial fibrillary acidic protein (GFAP) was identified as a prominent autoantigen recognized in all plasma samples. GFAP immunoreactivity was highest in DM areas and lowest in FC areas with no significant differences in anti-GFAP autoantibody titers between healthy controls and individuals with PD. The anti-GFAP autoimmunoreactivity of different brain areas correlates with the dissemination of histopathological neurodegeneration in PD. We hypothesize that GFAP autoantibodies are physiological but might be involved as a cofactor in PD pathogenesis secondary to a leakage of the blood–brain barrier.

## Introduction

Idiopathic Parkinson’s disease (PD) is characterized by a progredient neurodegeneration of the brain, starting at deep subcortical areas such as the dorsal motor nucleus of the glossopharyngeal and vagal nerves (DM) (stage 1), followed by the coeruleus–subcoeruleus complex; (stage 2), the substantia nigra (SN) (stage 3), the anteromedial temporal mesocortex (MC) (stage 4), high-order sensory association areas and prefrontal fields (HC) (stage 5) and finally first-order sensory association areas, premotor areas, as well as primary sensory and motor field (FC) (stage 6) (Braak et al 2003). The trigger for this ascending course of histopathological changes that is characterized by the presence of α-synuclein-immunopositive Lewy neurites and Lewy bodies (Braak et al 2003) remains unclear.

A number of studies suggested that autoimmune mechanisms, especially autoantibodies, might be involved in the pathogenesis of PD. Patients with PD presented with elevated serum levels of α-synuclein autoantibodies (Papachroni et al 2007) as well as GM1-ganglioside autoantibodies (Zappia et al 2002). Moreover, PD patients presented with a higher proportion of CSF-autoantibodies against substantia nigra homogenates than healthy controls (Carvey et al 1991). Autoantibodies directed at neuronal cells, brain lysates and dsDNA were also found to be more prevalent in patients with PD than healthy controls (Benkler et al 2002) as well as antibodies directed at neuromelanin (Double et al 2009). The CSF of PD patients was cytotoxic to cultivated dopaminergic neurons (Le et al. 1999) and IgG were found to be bound to neurons in histological examinations of brains from PD patients (Orr et al 2005). Brain-reactive autoantibodies were found to be ubiquitous in healthy individuals, suggesting that a leakage of the blood brain barrier (BBB) might be the trigger for autoimmune-mediated neuronal damage (Levin et al 2010). Finally, in an epidemiological study in Sweden with 310,000 subjects with 33 different autoimmune disorders, it was demonstrated that patients with autoimmune disease have 33% excess risk of developing PD (Li et al. 2012). In contrast, other studies did not find an association with immune disorders and PD (reviewed in Tan et al 2020).

Here we investigated the autoimmunoreactivity of different brain areas toward plasma samples from healthy individuals and patients with PD, with the aim to identify autoantigens that correlate with the histopathologic course of PD neurodegeneration in the brain.

## Materials and methods

### Ethics statement

The study was performed according to the declaration of Helsinki and approved by the ethics committee of the University of Würzburg, Germany (ethical clearance number 264/14).

### Plasma samples

Plasma samples were derived from the Neuro Biobank at the University Hospital in Tübingen, Germany. For this study, we analyzed 30 plasma samples from patients diagnosed with idiopathic Parkinson syndrome and 30 plasma samples of healthy controls.

### Brain tissue homogenates

Postmortem human brain tissue from cases with no clinical history of any neurological or neuropsychiatric disease plus confirmation by histopathological examination was obtained from Department of Neuropathology, University of Würzburg (member of BrainNet Germany). Whole human brains were obtained with the consent of the next of kin and according to the guidelines of the National and Local Ethics Committees. Causes of death were ascertained from clinical notes, autopsy reports or death certificates. In preparation for routine neuropathological examination, the brain was divided mid-sagittally and one hemisphere was immersed in 4.5% paraformaldehyde for 3–4 weeks. The remaining half was cryopreserved following coronal slicing for cortical sampling, sagittal slicing for sampling the cerebellum and transverse sectioning of the brain stem. Tissue samples were snap-frozen on brass plates, cooled in dry ice and stored at −80 °C until requested for experimental use. A neuropathological assessment was completed by a macroscopic and histological examination.

The following regions were isolated: dorsal motor nucleus of the glossopharyngeal and vagal nerves (DM), substantia nigra (SN), anteromedial temporal mesocortex (MC), high-order sensory association and prefrontal fields (HC), first or sensory association and premotor fields, primary sensoric and motoric fields (FC) according to Braak staging (Braak et al 2003). Tissues from three different donor brains were pooled homogenized on ice using a mechanical potter (Braun) in TE-buffer containing a protease inhibitor cocktail (Sigma). The protein concentration of the supernatants was determined by Bredford-test and adjusted to equal protein concentrations (1 µg/µl).

### SDS-PAGE and Western blotting

A total of 12 µg protein of each homogenates was loaded on precasted 4–12% gradient Tris-Glycin-SDS polyacrylamid gels (Invitrogen) and subjected to electrophoresis (35 mA, 125 V, 2 h). PageRuler™ Protein Ladder (Fermentas) was used as a molecular marker. The separated proteins were blotted on a nitrocellulose membrane using a tankblot system (Invitrogen). The membranes were stained with ponceau S solution to demonstrate equal protein load and equal protein transfer in all lanes. Membranes were blocked with PBS containing 5% low-fat milk-powder and 0.05% Tween-20 (TPBS) (Sigma). The membranes were cut into three pieces. Two pieces were incubated with the plasma sample or an internal control plasma that was used for all 60 blots (both diluted 1:50 in 2.5% milk-TPBS) over night at 4 °C, the third piece of the membrane was incubated in 2.5% milk-TPBS without any antibody. Membranes were washed and incubated with a secondary goat-anti-human IgG (light chain) antibody coupled to HRP (K3502, Sigma) in a dilution of 1:30,000. Membranes were incubated with enhanced ECL chemistry (Licor) and the Odyssee detection system (Licor).

2D-Gel electrophoresis and mass-spectroscopy-coupled protein sequencing was performed by a company (Proteome Factory AG, Berlin, Germany).

### Western blot quantification

Western Blot bands were densitometrically quantified using ImageStudioLite Software. For the comparison of band intensities in-between different brain areas, the relative intensities of the 45, 40 and 35 kD band intensities were calculated according to the following formula: [relative intensity of area *X*] = 100 × [sum of absolute intensities of 45, 40 and 35 kD bands of area *X*]/[sum of absolute intensities of 45, 40 and 35 kD bands of all area (DM, SN, MC, HC, FC)]. For the comparison of band intensities in-between different plasma samples, the relative single band intensities (i.e. either the 45 kD, the 40 kD or the 35 kD band) were calculated according to the formula: [relative intensity of band *X* in area *Y*] = [absolute intensity of band *X* in area *Y*]/[sum of absolute intensities of 45, 40 and 35 kD bands in the area DM of the internal control plasma].

#### ELISA

96-well microtiter plates were coated with 100 µl per well of 1 µg/ml recombinant human GFAP (ab114149, Abcam) in a 50 mM carbonate/bicarbonate buffer at pH 9.6 overnight. Plasma samples were diluted 1:50 in 2.5% milk-TPBS and incubated over night at 4 °C. The plate was washed three times with TPBS and incubated with a secondary goat-anti-human IgG (light chain) antibody coupled to HRP (K3502, Sigma) in a dilution of 1:30,000 in milk-TPBS for 1 h. Excess antibody was washed three times with TPBS. The ELISA reaction was run with a TMB substrate solution (Thermo Scientific) and sulfuric acid as a stop solution. The color reaction was quantified using a plate photometer at a wavelength of 450 nm.

### Statistical analysis

Experimental data were analyzed using the GraphPad Prism software. All data sets were analyzed for normal distribution of values using the D’Agostino & Pearson test. For two-group analyses, datasets were analyzed for statistical difference with the student’s *t*-test for normal distribution of values and with the Mann–Whitney *U*-test for non-normal distribution of values. For multi-group analyses, datasets were analyzed with one-way ANOVA (Friedmann test) for paired data sets for non-normal distribution of values. *X*/*Y*-correlations were analyzed by linear regression.

## Results

### Patient characteristics

We analyzed a total of 60 plasma samples from patients diagnosed with idiopathic Parkinson’s disease (30 samples) and neurologically healthy controls (30 samples) (Table 1) for the presence of anti-brain autoantibodies. The median age in the control sample group was 63 vs 65 years (difference statistically different, *p* = 0.0437). The proportion of females was 43.3% in the control group and 33.3% in the PD group (*p* = 0.5959).

**Table 1:** Patient characteristics

	Controls	PD	Level of significance
Number of plasma samples	30	30	n.a
Median age in years [range]	63 [56–71]	65 [56–76]	*p* = 0.0437^a^
Female sex in %	43.3%	33.3%	*p* = 0.5959^b^
Age at onset of PD [25–75% percentile]	n.a	55 [49.75–59.50]	n.a
Disease duration in years [25–75% percentile]	n.a	9.00 [6.00–13.25]	n.a
Hoehn & Yahr stadium [25–75% percentile]	n.a	2 [2–5]	n.a
UPDRS score [25–75% percentile]	n.a	0 [0–7]	n.a
MoCA score (of 30) [25–75% percentile]	n.a	28 [26–30]	n.a

^a^Student’s *t* test for values with normal distribution

^b^Mann–Whitney *U* test for values with non-normal distribution

### Detection of anti-brain autoantibodies

**Fig. 1:**
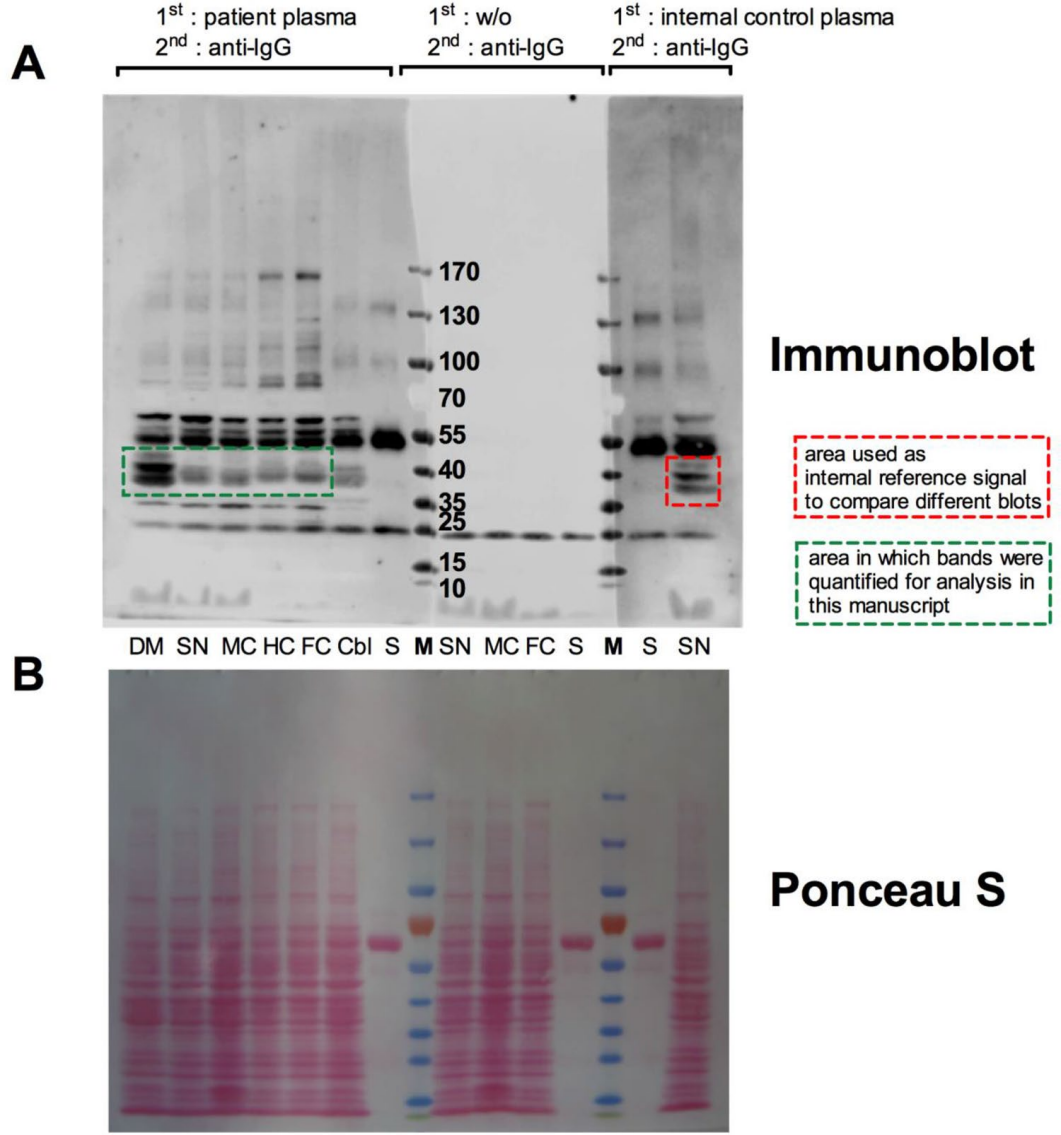
Anti-brain autoantibodies directed to different brain areas. Brain tissues from neurologically healthy deceased donors were dissected, homogenized, separated by SDS-PAGE and blotted on nitrocellulose membrane. Membranes were cut in three pieces and were incubated with plasma samples from patients with PD and healthy controls as first antibody and anti-human IgG as second antibody (left part of membrane depicted in **A**), with second antibody alone (middle part of membrane depicted in **A**) or with an internal control plasma sample of a healthy individual as first antibody and anti-human IgG as second antibody that was used for all 60 blots of this study (right part of membrane depicted in **A**). **B** Ponceau-S staining of the same membrane depicted in **A**, showing equal amounts of protein in each lane. Abbreviations: dorsal motor nucleus of the glossopharyngeal and vagal nerves (DM), substantia nigra (SN), anteromedial temporal mesocortex (MC), high-order sensory assoziation- and prefrontal fields (HC), first or sensory association- and premotor fields, primary sensoric and motoric fields (FC), cerebellum (Cbl), blood serum (S), marker (M)

**Fig. 2:**
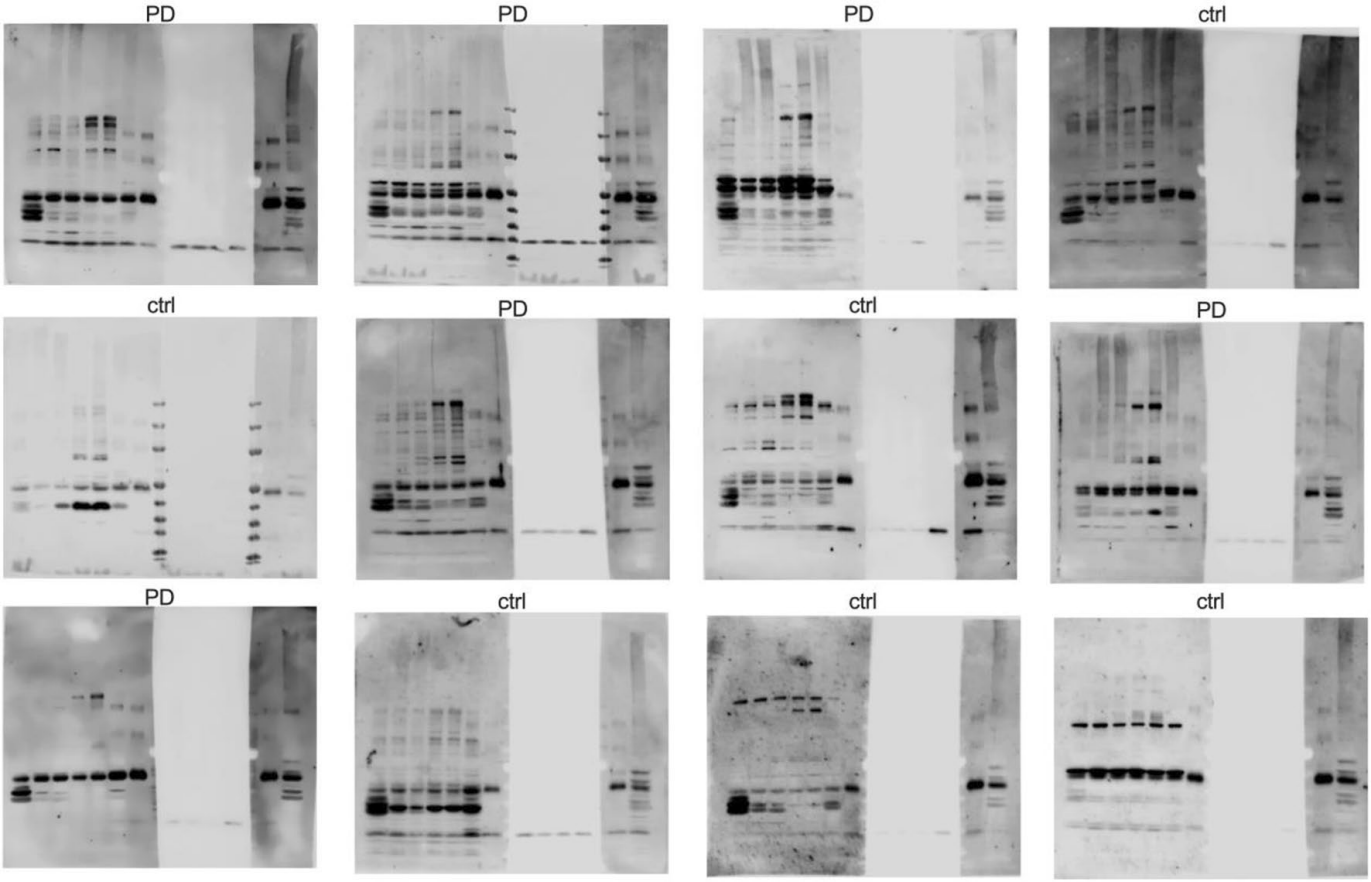
Representative collection of 12 Western Blots (of a total of 60) stained with plasma samples from healthy donors and patients with PD showing individual autoantibody patterns

The plasma from both healthy controls as well as PD patients showed immunoreactivity against the homogenates from different brain areas (Fig. 1A, shows a representative blot). All tested plasma samples recognized brain antigens as indicated by the occurrence of different bands. The patterns were different for each tested plasma sample (for illustration of the diversity of the patters see Fig. 2 with a comparison of 12 representative blots), indicating that different individuals have different amounts and specificities of autoantibodies recognizing different brain antigens. The absence of bands in the middle part of the blots that were only treated with secondary antibody but without plasma as first antibody shows that the bands on the left and right parts of the membrane are indeed specific for antibodies bound from the plasma samples and not from blood-derived antibodies from within the brain homogenates. The treatment with beta-mercaptoethaol within the SDS sample buffer dissolves most of the homogenate-derived antibodies into their heavy chains (50 kD) and light chains (25 kD). As we used an anti-light chain antibody as a secondary antibody, only the 25 kD-sized light chain band is to be seen in the middle part of the membrane, demonstrating that all other bands detected in the left and right part of the blot derive from bound antibodies originating from the plasma samples (if they originated from the homogenate, they would have shown up also in the middle part of the blot).

Most of the bands in the left and right parts originate from plasma antibodies recognizing brain tissue antigens and some other bands originate from plasma antibodies recognizing antigens derived from blood-plasma from within the homogenated tissue. To be able to tell which bands originate from truly tissue-specific antigens and which originate from blood-plasma proteins (mostly antibodies) within the tissue, we also loaded the gel with a serum sample (S). As seen in Fig. 1A, the serum lane predominantly displays a 50 and 25 kD band, corresponding to the beta-mercaptoethanol-reduced heavy- and light chains of the IgG, together with faint bands of approximately 150 kD (unreduced whole IgG) and approximately 100 kD (two still-connected heavy chains). If bands of this size also appear in the lanes of the tissue homogenates, it is very likely that these bands correspond to autoantibodies recognizing antibodies rather than tissue antigens. Bands of different sizes than 150, 100, 50 or 25 kD are therefore interpreted as autoantibodies recognizing brain tissue antigens. To be able to better compare band intensities between different blots for the analyses shown in Fig. 6, we used the right part of the membrane, which was always incubated with the same internal reference control serum for normalization: band intensities from the left part of the membrane (green box in Fig. 1A) were normalized to band intensities from the right side (red box in Fig. 1A) before comparison.

Despite individual differences between blots that made it very difficult to find a common pattern (Fig. 2), we noticed a prominent series of bands (35, 40 and 45 kD) especially in the DM preparation that showed up in many samples. We therefore decided to further characterize the antigen(s) contributing to these autoantibody bands (red box in Fig. 1A).

### Identification of the 45, 40 and 35 kD antigen

**Fig. 3:**
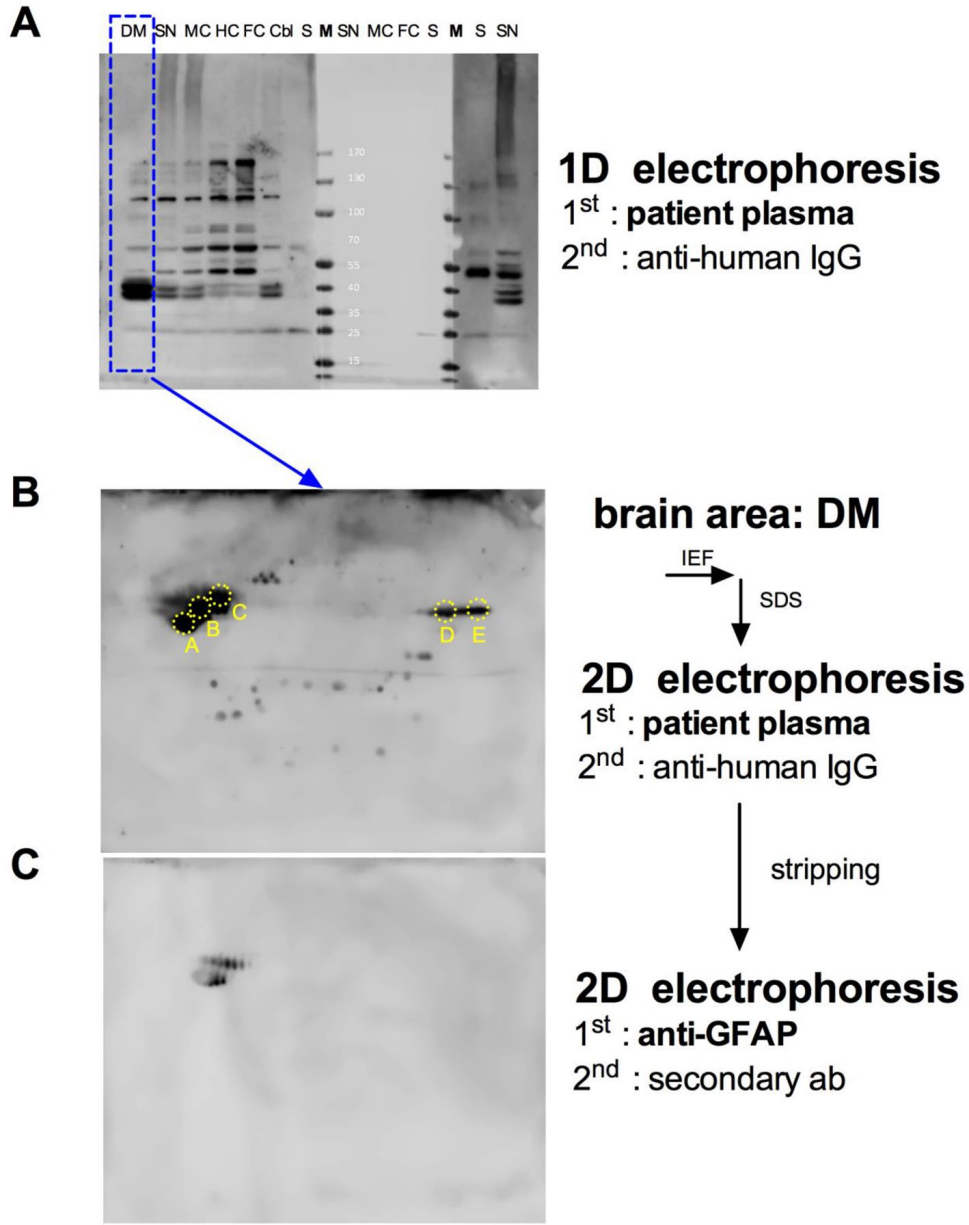
Identification of prominent autoantigens by 2D-Western Blot and MS-Sequencing. For the identification of the 45, 40, 35-kD cluster that is most prominent in the DM homogenate, DM homogenate was separated by 2D gel electrophoresis and blotted on a nitrocellulose membrane (**B**). The membrane was stained with a plasma sample that generated a strong 45, 40, 35 kD signal in the 1D Western Blot experiments (**A**). Proteins located at the strongest signals of the 2D-Western Blot were isolated and sequenced. The sequencing reaction identified the proteins underlying the spots A, B and C as GFAP (see also Table 2). **C** Reprobing of the membrane in B with anti-GFAP antibody. For abbreviations see Fig. 1

To identify candidates for the 35–45 kD antigens recognized by autoantibodies within most samples, we subjected the homogenate with the highest concentration of these antigens (DM) to a 2D-gel electrophoresis coupled with mass spectrometry (MS)-based sequencing of the proteins. Gels were run in parallel, one for the isolation of the proteins and subsequent MS-sequencing, one for Western Blotting to identify the localization of the antigens in the gel. We therefore stained the 2D-membrane with a plasma sample that generated a very strong and almost exclusive signal of the 35–45 kD proteins (Fig. 3A). The identified spots (Fig. 3B) were analyzed by MS-sequencing. The strongest signals (Spots A, B, C in Fig. 3B) corresponded to Glial fibrillary acidic protein (GFAP) isoforms 1 and 2 (Table 2). To test, whether GFAP is indeed located at the respective positions on the 2D gel, we reprobed the membrane depicted in Fig. 3B with an anti-GFAP antibody and detected GFAP at the same position (Fig. 3C).

**Table 2:** Proteins identified after mass spectrometry-sequencing from 2D gel electrophoresis

Spot	Sequence identity with highest score
A	GFAP Isoform 1
B	GFAP Isoform 2
C	GFAP Isoform 1
D	Fructose-Biphosphate aldolase A Isoform 1
E	Aspartate-Aminotransferase, mitochondrial isoform 1 precursor

Spots A–E are depicted in Fig. 3

### Confirmation of GFAP as the antigen behind the 35, 40 and 45 kD cluster

**Fig. 4:**
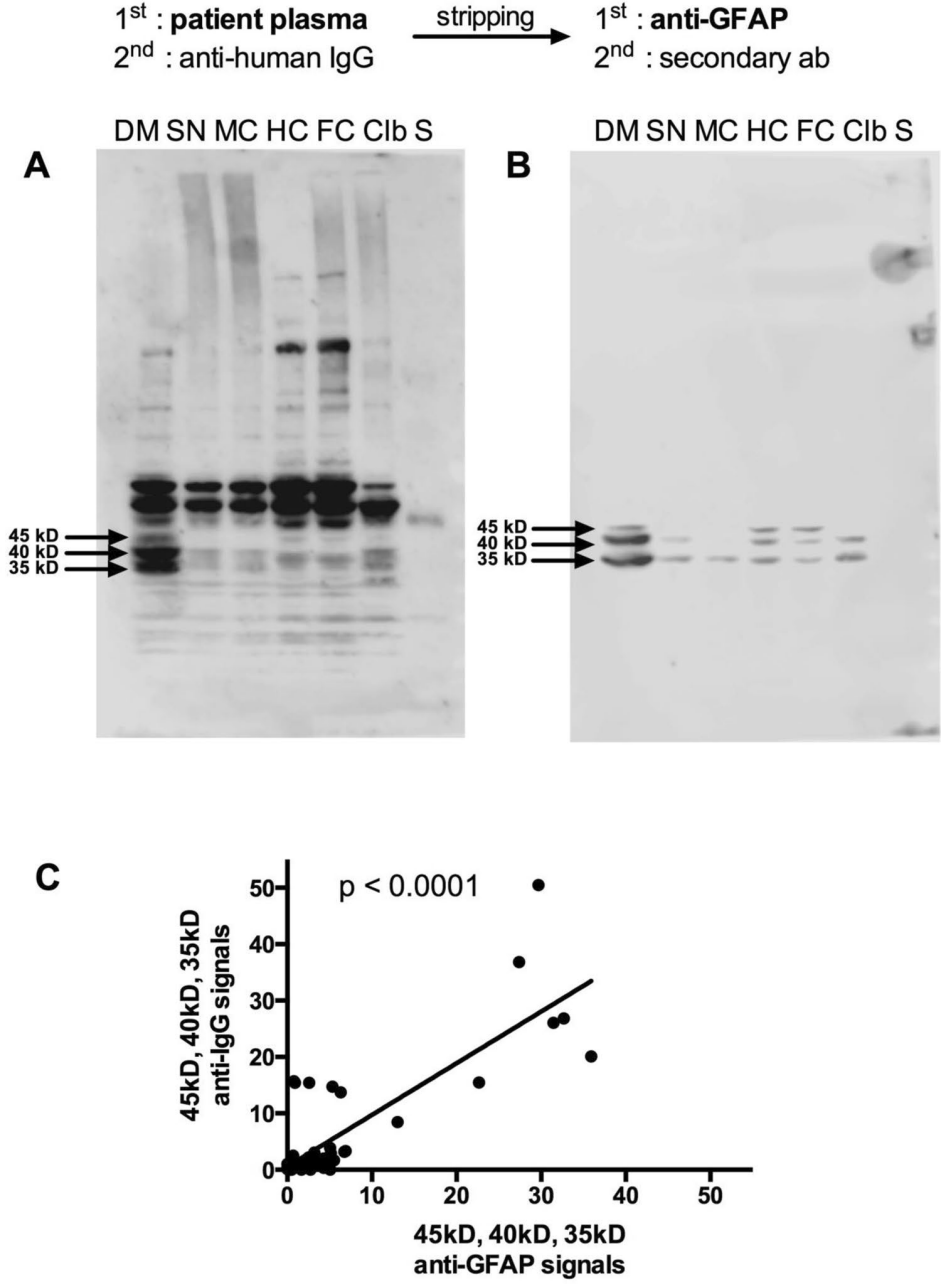
The prominent 35, 40, 45 kD autoantigens are identified as different GFAP isoforms. To test whether the 35, 40, 45 kD cluster seen in many plasma samples (**A**) is indeed caused by GFAP autoantibodies, the blot was reprobed with anti-GFAP antibody (**B**). Band Intensities from A and B were quantified and correlated (**C**) (*p* < 0.0001, linear regression). For abbreviations see Fig. 1

To confirm that the characteristic 35/40/45kD cluster detected in many plasma samples is indeed caused by autoantibodies recognizing GFAP, we reprobed three membranes that were previously probed with plasma samples with an anti-GFAP antibody (a representative blot is depicted in Fig. 4B). The GFAP antibody generated the same pattern of bands in the different lanes as the previous stain with the plasma samples. To test whether the anti-GFAP staining and the plasma sample-staining correlated with each other, we quantified the intensity of the bands and correlated the intensities of each bend with its corresponding partner band from the other stain. As depicted in Fig. 4C, the two signals exhibit a highly significant correlation (*p* < 0.0001), demonstrating that the 35, 40 and 45 kD bands that we quantify in this manuscript are indeed caused by autoantibodies recognizing GFAP as an autoantigen.

### GFAP is most prominently recognized in homogenates from brain areas with early pathology in PD

**Fig. 5:**
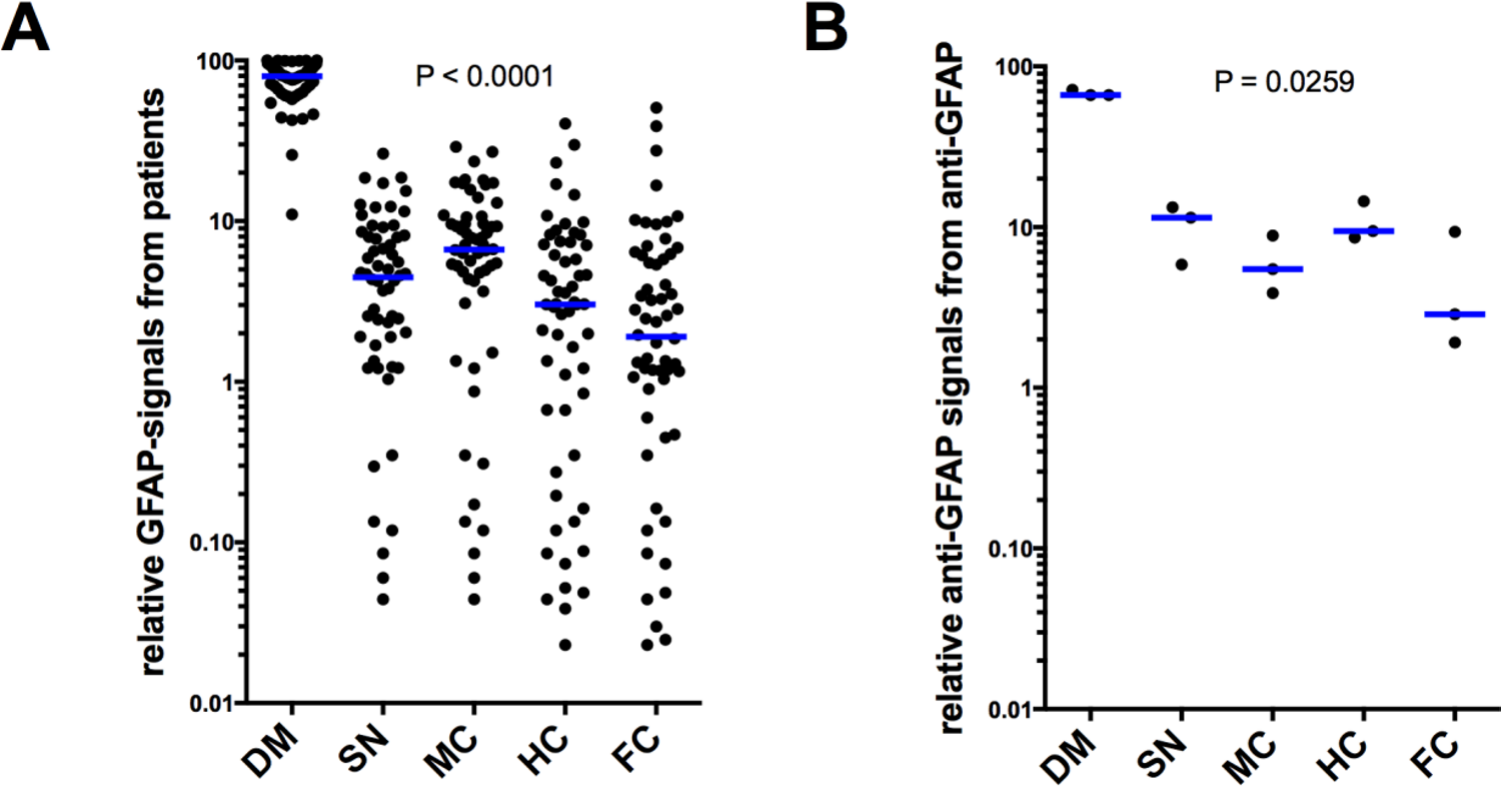
Anti-GFAP autoantibodies preferentially recognize brain areas involved in early PD pathology. The three GFAP-bands (35, 40, 45 kD) from all 60 samples were quantified (**A**). Three blots were reprobed with anti-GFAP (**B**), showing the same pattern (*p* calculated with ANOVA and Friedmann test). Abbreviations: dorsal motor nucleus of the glossopharyngeal and vagal nerves (DM), substantia nigra (SN), anteromedial temporal mesocortex (MC), high-order sensory assoziation- and prefrontal fields (HC), first or sensory association- and premotor fields, primary sensoric and motoric fields (FC)

To analyze whether the GFAP autoantibodies detected in most of the plasma samples differentially recognize different brain areas, we compared the band intensities (the sum of the 35, 40 and 45 kD bands) of the different brain areas (DM, SN, MC, HC, FC) generated by each plasma sample. For comparison, we set the sum of all three bands in all five regions to 100% for each Blot and displayed the relative signal intensities for each area and plasma sample (Fig. 5A). The area DM with the earliest onset of pathology in PD according to Braak et al. (2003) generates the most intense signal, whereas areas with later damage in PD generate weaker signals (*p* < 0.0001). To confirm that this pattern is not only to be seen in patient sera but also with an antibody recognizing GFAP, we reprobed three blots with an anti-GFAP antibody and got the same patters (Fig. 5B) (*p* = 0.0259).

### Comparison of the amount of anti-GFAP autoantibodies in plasma samples from PD patients and healthy controls

**Fig. 6:**
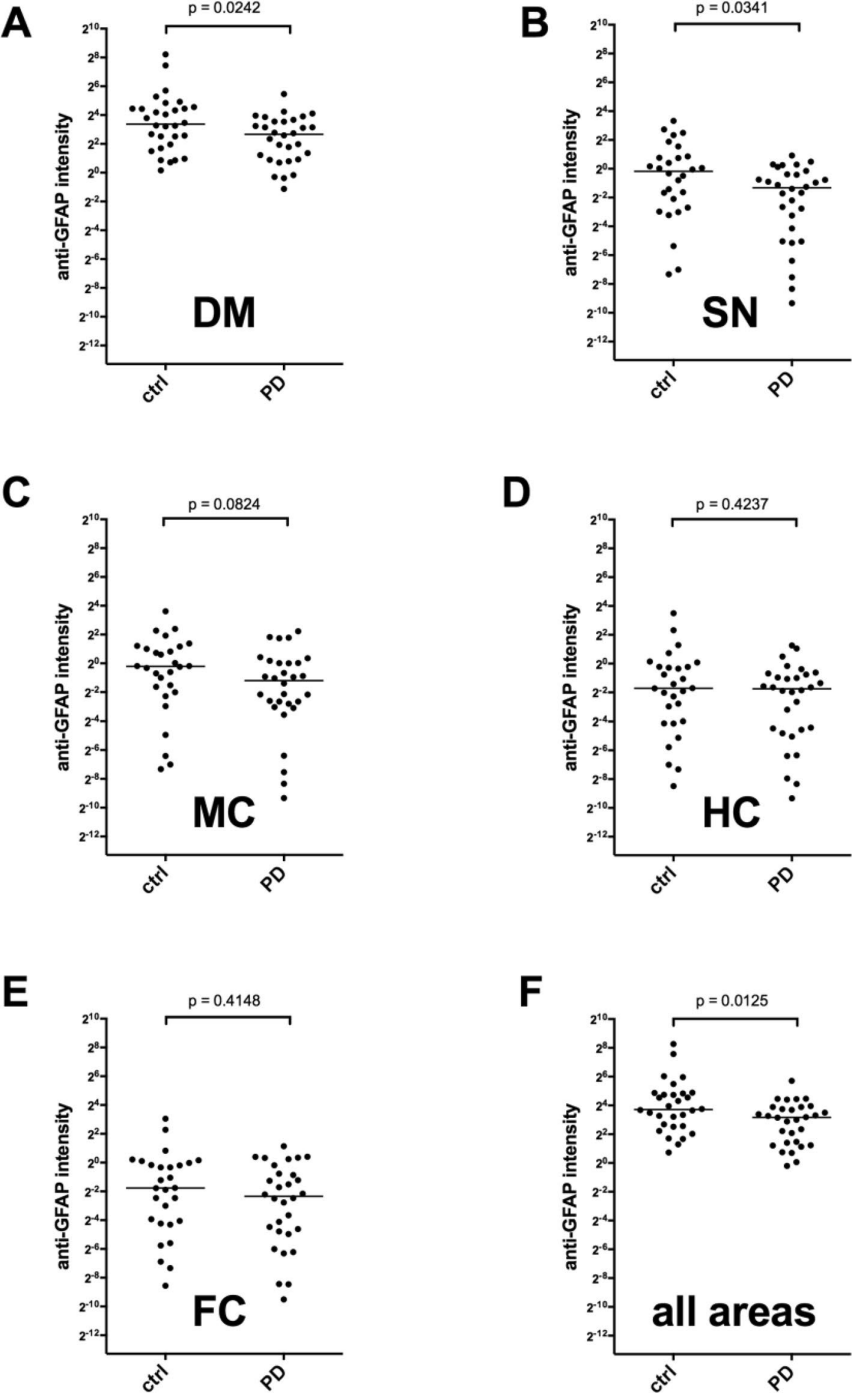
Comparison of anti-GFAP autoantibody titers of healthy controls and PD patients. Sum of the 35, 40, 45 KD GFAP bands for each brain area relative to the internal standard plasma (*p* calculated with Mann–Whitney *U*-test). Abbreviations: dorsal motor nucleus of the glossopharyngeal and vagal nerves (DM), substantia nigra (SN), anteromedial temporal mesocortex (MC), high-order sensory assoziation- and prefrontal fields (HC), first or sensory association- and premotor fields, primary sensoric and motoric fields (FC)

**Fig. 7:**
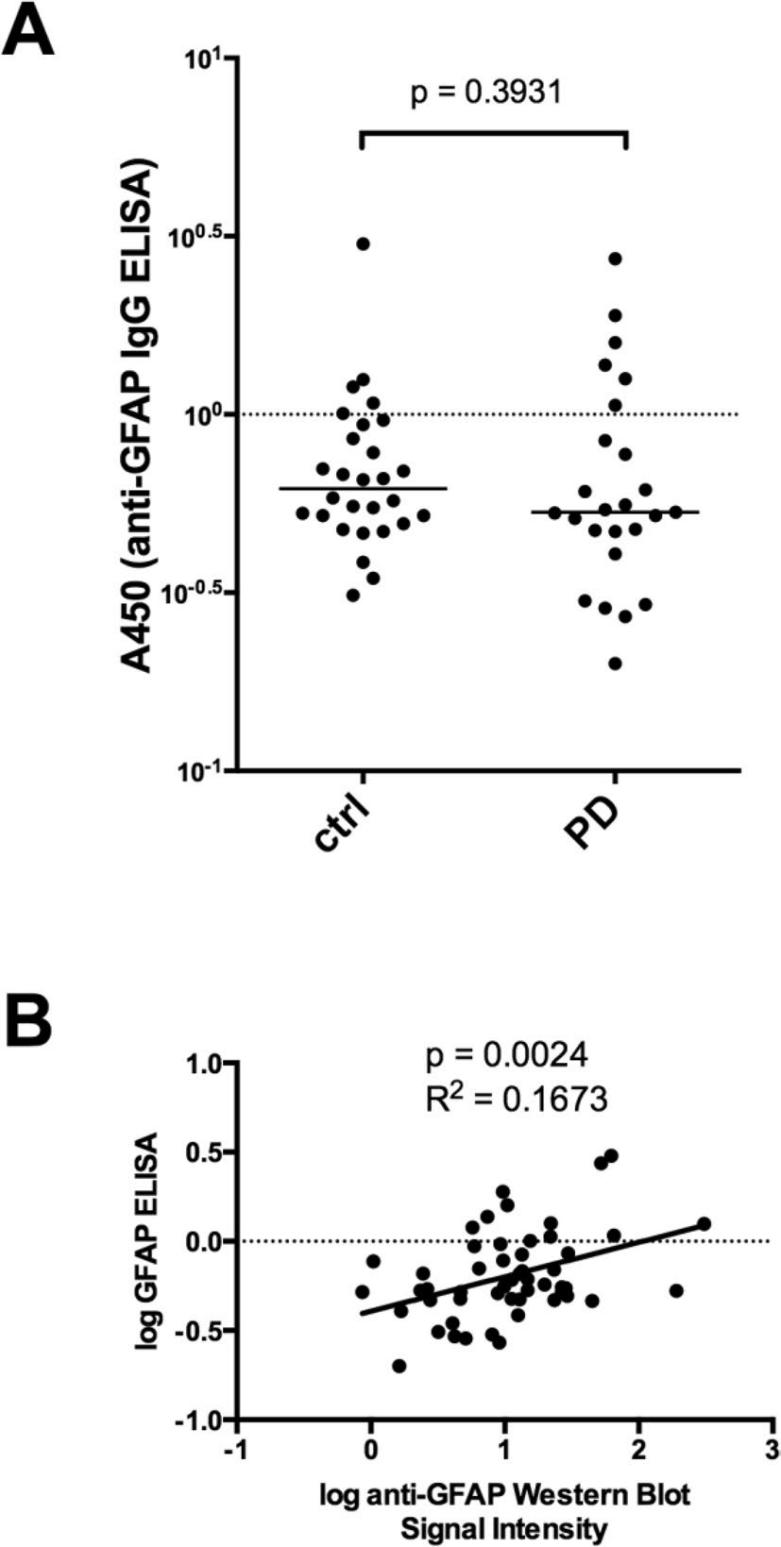
Quantification of anti-GFAP autoantibodies with an ELISA. **A** GFAP autoantibody concentrations of the 60 plasma samples were determined by ELISA. **B** Linear correlation of the signals of the 35, 40, 45 kD band signal from Fig. 6F with the ELISA data from **A**

We used the semiquantitative data from the Western Blots also for a comparison of the anti-GFAP autoantibody titers in the plasma samples from PD patients and healthy controls. The antibody titers in the PD samples were a little lower than the titers detected in the control group (Fig. 6). Although this difference reached statistical significance (*p* = 0.0125 for all tested areas, Fig. 6F), the detected difference was only in the range of a factor of 2. It therefore seems questionable whether this difference, although statistically significant, is of any clinical relevance. Moreover, the signal intensities between different blots probed with identical plasmas also vary in the range of a factor of 2 for our assay system (data not shown), so that we decided to set up an ELISA detecting anti-GFAP autoantibodies to better quantify the GFAP autoantibody titers in our sample groups. We therefore coated an ELISA plate with recombinant human GFAP, incubated it with diluted plasma samples (1:50) and detected bound antibody with an anti-IgG antibody. As shown in Fig. 7A, no difference in anti-GFAP autoantibody titers between the two groups could be detected. Overall, the ELISA data correlated with the Western Blot data (Fig. 7B, *p* = 0.0024), but the variance of the data was relatively high (*R*^2^ = 0.1673).

## Discussion

Here, we have shown that autoantibodies against human brain tissue and in particular against GFAP are detectable in almost all investigated plasma samples, independent of whether the samples were derived from healthy controls or from patients with PD. Anti-GFAP autoantibody levels were similar between samples from PD patients and healthy controls. Different areas from post mortem brains are recognized differently by these anti-GFAP autoantibodies, with the highest immunoreactivity detected against homogenates from the dorsal motor nucleus of the glossopharyngeal and vagal nerves (DM) and the lowest immunoreactivity detected against cortical regions (HC, FC). The median autoimmunoreactive difference between the area with the highest immunoreactivity (DM) and the area with the lowest immunoreactivity (FC) is in the range of almost two log scales, and reached statistical significance (*p* < 0.0001).

According to a careful and systematic investigation of histopathological changes in postmortem brains from deceased patients with PD (Braak et al. 2003), the dorsal motor nucleus of the glossopharyngeal and vagal nerves (DM) is the first brain region to be affected in PD (stage 1), followed by the coeruleus–subcoeruleus complex; (stage 2), the substantia nigra (SN) (stage 3), the anteromedial temporal mesocortex (MC) (stage 4), high-order sensory association areas and prefrontal fields (HC) (stage 5) and finally first-order sensory association areas, premotor areas, as well as primary sensory and motor field (FC) (stage 6) (Braak et al 2003). The anti-GFAP autoimmunoreactivity described in our manuscript follows this path (unfortunately we have not been able to include coeruleus tissue into our investigation), suggesting that anti-GFAP autoantibodies might be involved in PD pathogenesis: our findings support the hypothesis that anti-GFAP autoantibodies may acquire pathophysiological significance under certain conditions, such as transient permeability of the blood–brain barrier (Levin et al 2010). Under normal conditions, plasma antibodies are excluded from the brain by the blood brain barrier (BBB), but a breakdown of its integrity might render the brain susceptible for an autoimmune attack of these autoantibodies.

It has been reported that autoantibodies against GFAP are found in a recently recognized pathological entity, the autoimmune GFAP astrocytopathy (Shan et al. 2018), and/or develop after traumatic brain injury (Zhang et al. 2014; Wang et al. 2016). In this context, it is noteworthy that head trauma is also a long-known risk factor in PD (Stern1991). GFAP is almost exclusively expressed in the brain, predominantly in astrocytes, as a 50 kD protein (reviewed in Yang and Wang 2015) with typical calpain-mediated truncations in the range of 38–48 kD when analyzed in Western Blot (Zhang et al 2014). GFAP-positive cells have been detected at high density in the dorsal motor nucleus compared to other brainstem structures (Pecchi et al 2007). Astrocytes, presumably the main target cell of an anti-GFAP autoimmune reaction, play a vital role in multiple processes in the brain, including synapse formation, clearance of neurotransmitters, or modulation of synaptic activity and plasticity (reviewed in Vasile et al 2017), and an anti-GFAP autoimmunity, as reported in our manuscript, might therefore very well be linked to several neurological disorders as well to the specific autoimmune GFAP astrocytopathy (Kunchok et al 2019). Moreover, recently a case of GFAP-astroglial autoimmunity presented with reversible parkinsonism (Tomczak et al 2019), suggesting the close relationship between these entities.

We acknowledge some limitations of our study: The sample size in this study (60 in total with 30 vs 30 from patients with PD vs controls) is relatively low and therefore, the study is especially vulnerable to sampling effects. The here-reported comparison of anti-GFAP autoantibody titers between PD and control samples should therefore be reexamined by subsequent studies with larger sample sizes. This is even more so as the patient characteristics in our study reveal a significant difference in age between the two groups (63 years for controls vs. 65 years for PD patients, *p* = 0.0437, Table 1) and increasing age is a factor that usually correlates with lower antibody titers. When we do a correlation between age and anti-GFAP signal with the samples in this study, we see a trend toward slightly lower GFAP-titers with increasing age (*p* = 0.1205, data not shown). Another limitation of our study is that astrocyte numbers and activation state may vary in different brain regions over time. For example, astrocyte density in the brain changes with age (Nichols et al. 1993) or neurodegenerative diseases including PD (Damier et al. 1993). By the strategy of pooling homogenates from three different donor brains used in our manuscript, we tried to equalize such potential intra- and inter-individual differences. However, it will be interesting to investigate in further studies possible differences in GFAP expression, especially in the dorsal motor nucleus, in more detail by comparing different donor homogenates, and also extending the study to brains from deceased PD patients.

We have shown that autoantibodies against GFAP are found at high levels in many individuals. GFAP autoantibodies are found equally in patients with idiopathic PD and healthy controls, so that no direct correlation between the presence of these autoantibodies and the pathogenesis of PD can be deduced. However, brain areas that are normally destroyed early in the pathogenesis of PD, such as the dorsal motor nucleus, have particularly high levels of GFAP. This leads to the hypothesis that disturbances in the integrity of the blood–brain barrier could lead to an access of these autoantibodies to the particularly GFAP-rich areas, so that these autoantibodies could then acquire a pathological function in the development of PD.
